# Top-Down Proteomic Identification of Shiga Toxin 1 and 2 from Pathogenic *Escherichia coli* Using MALDI-TOF-TOF Tandem Mass Spectrometry

**DOI:** 10.3390/microorganisms7110488

**Published:** 2019-10-25

**Authors:** Clifton K. Fagerquist, William J. Zaragoza, Michelle Q. Carter

**Affiliations:** Produce Safety & Microbiology, Agricultural Research Service, U.S. Department of Agriculture, 800 Buchanan Street, Albany, CA 94710, USA; william.zaragoza@usda.gov (W.J.Z.); michelle.carter@usda.gov (M.Q.C.)

**Keywords:** Shiga-toxin-producing *Escherichia coli*, STEC, MALDI-TOF-TOF, mass spectrometry, tandem mass spectrometry, MS/MS, antibiotic induction, aspartic acid effect, disulfide bond, top-down proteomics

## Abstract

Shiga-toxin-producing *Escherichia coli* (STEC) are a burden on agriculture and a threat to public health. Rapid methods are needed to identify STEC strains and characterize the Shiga toxin (Stx) they produce. We analyzed three STEC strains for Stx expression, using antibiotic induction, matrix-assisted laser desorption/ionization time-of-flight-time-of-flight (MALDI-TOF-TOF) mass spectrometry, and top-down proteomic analysis. *E. coli* O157:H- strain 493/89 is a clinical isolate linked to an outbreak of hemolytic uremic syndrome (HUS) in Germany in the late 1980s. *E. coli* O145:H28 strains RM12367-C1 and RM14496-C1 were isolated from an agricultural region in California. The *stx* operon of the two environmental strains were determined by whole genome sequencing (WGS). STEC strain 493/89 expressed Shiga toxin 2a (Stx2a) as identified by tandem mass spectrometry (MS/MS) of its B-subunit that allowed identification of the type and subtype of the toxin. RM12367-C1 also expressed Stx2a as identified by its B-subunit. RM14496-C1 expressed Shiga toxin 1a (Stx1a) as identified from its B-subunit. The B-subunits of Stx1 and Stx2 both have an intramolecular disulfide bond. MS/MS was obtained on both the disulfide-bond-intact and disulfide-bond-reduced B-subunit, with the latter being used for top-down proteomic identification. Top-down proteomic analysis was consistent with WGS.

## 1. Introduction

Shiga-toxin-producing *Escherichia coli* (STEC) continue to be linked to major outbreaks of foodborne illness worldwide [1,2]. Shiga toxin (Stx) is an AB5 toxin comprised of five identical B-subunits and one catalytically-active A-subunit. These proteins assemble into a non-covalent quaternary complex, where the B-subunits form a donut-shaped structure having five-fold symmetry. Most of the A-subunit is positioned on one side of the “donut”, with a part of it fitting within the “donut” hole.

The Stx holotoxin attaches to surface receptors of eukaryotic cells (e.g., globotriasylceramide or Gb3). Holotoxin attachment occurs on the side opposite to that of the A-subunit, primarily as a result of the tertiary and quaternary structures of the five B-subunits. Each B-subunit has an intramolecular disulfide bond that is critical to its secondary and tertiary structure. The B-subunits play a critical role in the successful attachment of the holotoxin to eukaryotic cells. The attached holotoxin is enveloped by the eukaryotic cell by endocytosis. It then follows a retrograde pathway from the early endosome to the Golgi to the endoplasmic reticulum (ER) and finally to the cytoplasm, where it disrupts protein synthesis. Proteolytic cleavage of the A-subunit at an exposed loop in the polypeptide chain, followed by reduction of a disulfide bond at the base of the loop, releases the A1 protein fragment into the cytoplasm, where it disables the ribosomal machinery of protein synthesis, leading to cell death [3].

There are two major Stx types: Stx1 and Stx2. The amino acid sequence of Stx1 and Stx2 are similar but different, with about 50% homology [4]. Stx1 and Stx2 are further classified into subtypes: a, c, and d for Stx1, and a–g for Stx2 [5]. All Stx1 subtypes are clinically relevant, whereas, for Stx2, subtypes a–d are clinically relevant and subtypes e–g are not clinically relevant. The toxicity of Stx has been found to vary with amino acid sequence, suggesting that slight changes in the amino acid sequence can have a significant effect on holotoxin/receptor attachment. For example, Stx2 has been more often linked to major outbreaks of foodborne illness, and these outbreak STECs often produce Stx2a [6,7,8]. Methods to distinguish types and subtypes of Stx have significant utility.

*Stx* genes are in the genome of prophages present in the bacterial-host genome. The spread of *stx* genes across bacteria is due to horizontal transfer of its genes when the lytic cycle of the prophage is initiated. Initiation of the lytic cycle can be caused by environmental stresses, e.g., starvation stress (or nutrient deprivation), antibiotic stress, UV exposure, etc. [9]. Expression of prophage genes, including *stx*, is often induced by antibiotic stress or nutrient-deprivation stress of a putative STEC strain.

After microbiological isolation of a putative STEC strain, its pathogenic potential is usually evaluated, using either polymerase chain reaction (PCR) or whole-genome sequencing (WGS) to determine the presence of pathogenic genes [5,10,11]. Assays can also be performed to detect expression of the Stx, using latex-agglutination assay or enzyme-linked immunoassay (ELISA) [12,13]. Bioassays have also been developed to measure the Stx toxicity by its disruption of protein synthesis in mammalian cells cultured in vitro [14,15].

Mass spectrometry and mass-spectrometry-based proteomic techniques have distinct advantages for the detection and identification of peptides, proteins, and other biomolecules. Tandem mass spectrometry (MS/MS) can provide sequence-specific information. Mass-spectrometry-based proteomic techniques may involve bottom-up or top-down proteomic analysis. Bottom-up involves digestion of the protein, followed by analysis of its constituent peptides, usually by liquid chromatography (LC)-MS/MS. Top-down involves MS/MS analysis of the mature, intact protein, with no protein digestion. Both top-down and bottom-up have advantages and disadvantages, and both have been used in the analysis of Stx [16,17,18].

In the current study, we have used antibiotic induction, matrix-assisted laser desorption/ionization time-of-flight-time-of-flight (MALDI-TOF-TOF) MS/MS, and top-down proteomic analysis to examine three STEC strains. Two strains are environmental isolates from an agricultural region in California, and the third is a sorbitol-fermenting clinical isolate linked to an outbreak of hemolytic uremic syndrome (HUS) in Europe in the late 1980s.

## 2. Materials and Methods

### 2.1. Bacterial Strains and Culturing

The bacterial strains used in this study are shown in Table 1 [19,20,21]. Shiga-toxin-producing *E. coli* (STEC) are pathogenic bacteria. All microbiological manipulations were performed in a Class 2 biohazard safety cabinet. STEC strains were cultured in Luria-Bertani (LB) broth from glycerol stocks for four hours, with shaking 250 rpm at 37 °C. An aliquot of cells was spread onto an LB agar plate supplemented with antibiotic (i.e., ciprofloxacin or mitomycin-C) and grown overnight, statically, at 37 °C. After culturing, the plates were examined for bacterial growth.

The optimum antibiotic culturing concentration for Stx expression was found to be strain-dependent. Initially, each strain was cultured on LBA supplemented with 10 and 20 ng/mL of ciprofloxacin and 800 and 1200 ng/mL of mitomycin-C. After overnight culturing, the plates were then examined for bacterial growth and analyzed by MALDI-TOF-MS for B-subunit detection. If the optimum antibiotic concentration for Stx production for a strain had growth that was inadequate to fill a 1 µL loop of cells, more plates were subsequently cultured at the optimum concentration, to harvest enough cellular material.

Cellular material was harvested from the plate with a 1 µL loop and transferred to 300 µL of water in a microcentrifuge tube with a screw-cap lid. The tube was briefly vortexed and then centrifuged at 14,000× *g* for 2 min, as previously described [22]. A 0.60 µL aliquot of the supernatant was spotted onto the 384-spot stainless steel MALDI target and allowed to dry. The dried sample spot was then overlaid with 0.75 µL aliquot of a saturated solution of MALDI matrix: 33% acetonitrile, 67% water, and 0.2% trifluoroacetic acid and 10 mg of sinapinic acid (ThermoFisher, San Jose, CA, USA), which was also allowed to dry. For disulfide reduction, 1 µL of 1.0 M dithiothreitol solution (Sigma-Aldrich, St. Louis, MO, USA) was added to 30 µL of supernatant and heated for 10 min, at 70 °C, in a water bath. The reduced sample was then similarly spotted onto the MALDI target plate. The MALDI plate was then inserted into the mass spectrometer for analysis.

### 2.2. Mass Spectrometry Analysis

Mass spectrometry analysis was performed using a 4800 MALDI-TOF-TOF mass spectrometer (Sciex, Redwood City, CA) equipped with a pulsed solid-state YAG laser (λ = 355 nm, 200 Hz repetition rate). The instrument was externally calibrated in MS linear-mode, using the +1 and +2 charge states of lysozyme, cytochrome-C, and myoglobin (Sigma-Aldrich, St. Louis, MO, USA). The instrument was externally calibrated in reflectron-mode for tandem mass spectrometry (MS/MS), using five fragment ions of alkylated thioredoxin (AlkTrx), as described previously [23].

MS and MS/MS data were collected using the instrument control software (4000 Series Explorer, Version 3.5.3, Sciex, Redwood City, CA, USA). Sample spots were analyzed first in MS linear-mode, acquiring 1000 laser shots (5 s acquisition). The laser fluence was adjusted to slightly above threshold (~5000 au). After the desorption/ionization laser pulse and a time delay, ions were accelerated from the source at 20.0 kV, after which they were spatially separated into discrete ion packets (that differ in *m/z* and velocity) in the flight tube, until they struck the linear detector. Multiple technical replicates were acquired to confirm protein-ion detection.

The protein ions detected in linear-mode were then analyzed in MS/MS reflectron-mode. No target gas was used in the collision cell. Precursor ions were fragmented by post-source decay (PSD) [24]. In this mode, ions were accelerated from the 1st source at 8.0 kV, the analyte ion was isolated by the timed-ion selector (TIS), and then de-accelerated to 1.0 kV, prior to entering the collision cell. After exiting the collision cell, ions were re-accelerated to 15.0 kV. Unfragmented precursor ion was deflected off the ion path by the metastable suppressor. Fragment ions were separated by the two-stage reflectron mirror, where they were reflected nearly 180° toward the reflectron detector.

In order to obtain the maximum amount of protein-ion fragmentation, the laser fluence was adjusted to 7500–7900 au to increase the amount of energy deposited in the precursor ions for subsequent fragmentation in the collision cell. However, one consequence of using such high laser fluence was that the matrix spot was quickly exhausted. For a single MS/MS experiment, 10,000 laser shots (50 s) were acquired, after which the MALDI spot was exhausted.

### 2.3. Gene and Genomic Sequencing

The *stx* operon of *E. coli* O157:H- strain 493/89 was previously sequenced [19]. The *stx* operon of *E. coli* O145:H28 strains RM12367-C1 and RM14496-C1 were obtained by single-molecule real-time (SMRT) sequencing on a PacBio RSII instrument (Pacific Biosciences, Menlo Park, CA, USA), as described previously [25].

### 2.4. Top-Down Proteomic Analysis

Data were viewed, processed, and exported using Data Explorer^®^ (Version 4.9, Sciex, Redwood City, CA, USA). MS data were unprocessed. MS/MS data were processed in the following sequence of steps: advanced baseline correction (peak width: 32, flexibility: 0.5, degree: 0.0), followed by noise removal (standard deviation: 2), followed by Gaussian smoothing (filter width: 31 pts). For top-down proteomic analysis, processed MS/MS data were centroided and exported as an ASCII data file containing two columns (*m/z* and absolute intensity) of fragment ion data. The ASCII file was then uploaded to the database of our in-house top-down proteomic software [26]. Identifications were reported in both the percentage of matched fragment ions (USDA score) and *p*-values [26,27].

## 3. Results and Discussion

### 3.1. Analysis of E. coli O157:H- Strain 493/89

**Figure 1:**
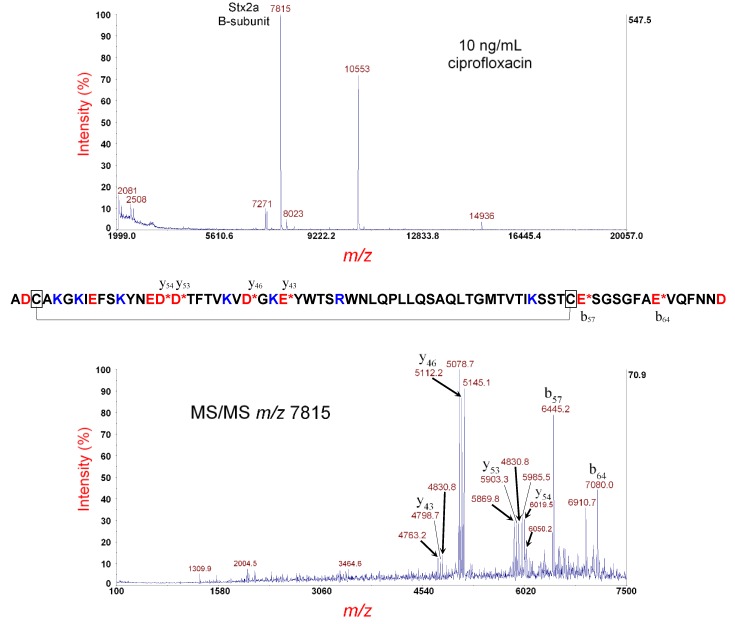
Top panel: MS analysis of *E. coli* O157:H- strain 493/89 cultured overnight on LBA supplemented with 10 ng/mL of ciprofloxacin. The putative disulfide-bond-intact B-subunit of Stx2a is indicated. Bottom panel: MS/MS analysis of the putative B-subunit at *m/z* 7815 in the top panel. Fragment ions are indicated by their *m/z* and b- or y-type designations. The sequence of the Stx2a B-subunit is shown. A red asterisk indicates sites of polypeptide backbone cleavage that generate the corresponding fragment ions shown above (y-type) or below (b-type) the sequence. Acidic residues are highlighted in red and basic residues in blue. Cysteine residues are boxed, and a connector line symbolizes the intramolecular disulfide bond.

Figure 1 (top panel) shows the MS analysis of the *E. coli* O157:H- strain 493/89 cultured overnight on LBA supplemented with 10 ng/mL of ciprofloxacin. The peak at *m/z* 7815 is the [M+H]^+^ of the putative B-subunit of Stx2a. The theoretical average mass of the disulfide-bond-intact (oxidized) B-subunit is 7815.62 Da. The difference in mass between the observed and theoretical values is within experimental error for MS linear-mode analysis (1000 ppm). A few bacterial host and prophage proteins were also detected at *m/z* 7271, 10553, and 14936.

The putative Stx2a B-subunit ions at *m/z* 7815 were isolated and fragmented by MS/MS-PSD. The results are shown in Figure 1 (bottom panel), where prominent fragment ions are identified by their *m/z* and b- or y-type fragment ion designation. The mature amino acid sequence of the B-subunit (without its N-terminal signal peptide) is shown above. A red asterisk (*) denotes the site of polypeptide backbone cleavage. Basic residues are highlighted in blue and acidic residues in red. Cysteine residues are boxed with a line connecting them to symbolize the intramolecular disulfide bond. The MS/MS data show that the most prominent fragment ions are the result of polypeptide backbone cleavage on the C-terminal side of aspartic acid (D) and glutamic acid (E) residues, and this is known as the aspartic acid effect [24,28]. For polypeptide backbone cleavage between the two cysteine residues involved in the intramolecular disulfide bond, a trio of fragment ions (separated by ±33 Da) is detected, resulting from symmetric and asymmetric cleavage of the disulfide bond [29]. Fragment ion triplets (e.g., *m/z* 5078.7, 5112.2, and 5145.1) are the result of polypeptide backbone cleavage between the two cysteine residues and symmetric (S···S) and asymmetric cleavage (-SSH and HSS-) of the disulfide bond. Polypeptide backbone cleavage not between the two cysteine residues does not show fragment ion triplets as expected. The large lasso-looped structure of the disulfide-bond-intact B-subunit appears to favor a nearly equal probability of the three dissociation channels for disulfide bond rupture.

**Figure 2:**
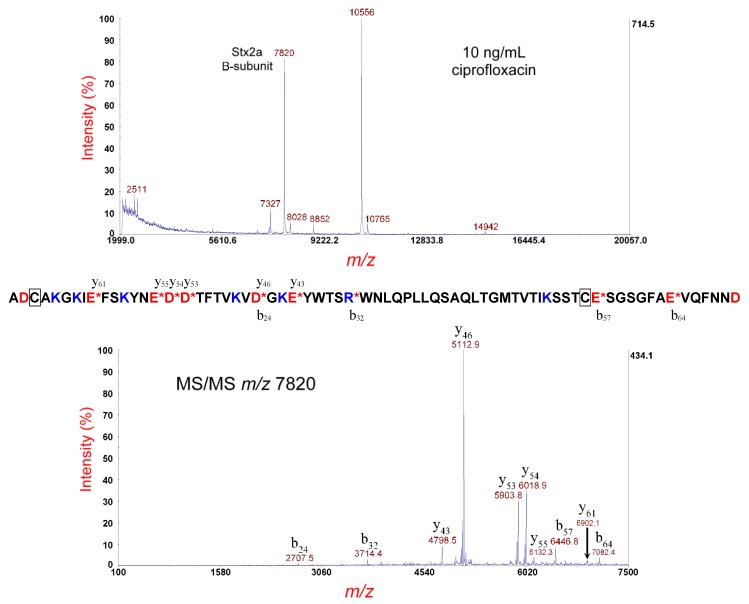
Top panel: MS analysis of *E. coli* O157:H- strain 493/89 cultured overnight on LBA supplemented with 10 ng/mL of ciprofloxacin. The sample supernatant was disulfide-reduced. The putative disulfide-reduced B-subunit of Stx2a is indicated. Bottom panel: MS/MS analysis of the putative B-subunit at *m/z* 7820 (top panel). Fragment ions are indicated by their *m/z* and b- or y-type designations. The sequence of the Stx2a B-subunit is shown. A red asterisk indicates sites of polypeptide backbone cleavage that generate the corresponding fragment ions shown above (y-type) or below (b-type) the sequence. Acidic residues are highlighted in red and basic residues in blue. Cysteine residues are boxed.

Figure 2 (top panel) shows MS analysis of the same strain cultured under the same conditions; however, the sample was reduced prior to MS analysis. A peak at *m/z* 7820 is the [M+H]^+^ of the putative disulfide-reduced B-subunit of Stx2a. The theoretical average mass of the disulfide-bond-reduced B-subunit is 7817.64 Da; thus, the *m/z* is within experimental error. The putative reduced B-subunit at *m/z* 7820 was isolated and fragmented by MS/MS-PSD, and the resulting spectrum is displayed in Figure 2 (bottom panel). We observe a significant change in the relative intensity of the fragment ions detected compared to the same protein ion with the disulfide bond intact. The fragment ion triplets are now completely absent, as one would expect, with the reduction of the disulfide bond. Unlike the large lasso-loop structure of the disulfide-bond-intact B-subunit, a disulfide-reduced B-subunit is now a linear polypeptide chain, resulting in a much less complicated MS/MS spectrum. The most prominent fragment ions are still on the C-terminal side of D- and E-residues. It is interesting to observe that b_57_ and b_64_ (from cleavage on the C-terminal side of E-residues) are quite prominent in the MS/MS spectrum of the disulfide-intact B-subunit but are relatively weak in the disulfide-reduced B-subunit. For the disulfide-intact B-subunit, b_57_ and b_64_ result from cleavage of the short peptide “tail” attached to the larger polypeptide “loop”, whereas, in the disulfide-reduced B-subunit, the dissociation channels that generate b_57_ and b_64_ must now compete with the more efficient dissociation channels on the C-terminal side of D-residues of the linear polypeptide chain structure.

The disulfide-bond-reduced B-subunit is used for top-down proteomic identification because of the simplicity of its MS/MS-PSD fragmentation. Top-down proteomic analysis, using our in-house proteomic software, identified this protein as the Stx type/subtype: Stx2a with a *p*-value of 4.8 × 10–15 and a percentage of matched fragment ions: 40.48 (USDA score) [26,27]. The results were consistent with genomic sequencing.

### 3.2. Analysis of E. coli O145:H28 Strain RM12367-C1

**Figure 3:**
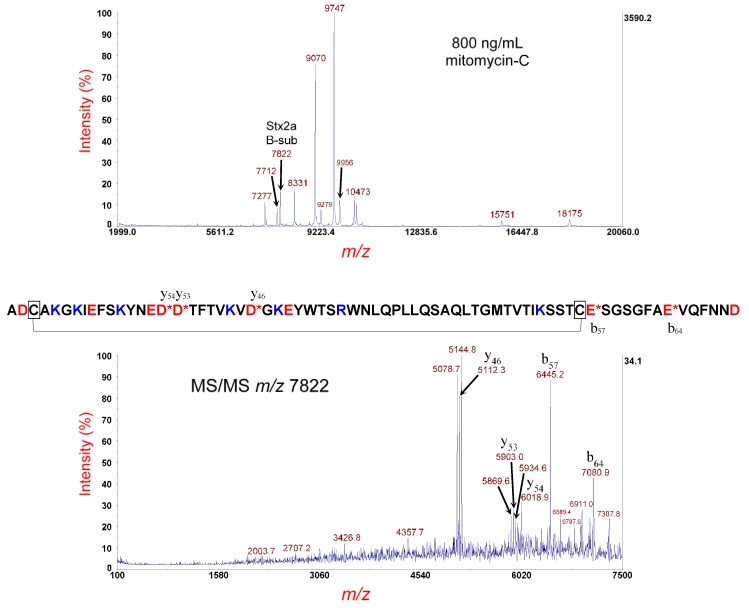
Top panel: MS analysis of *E. coli* O145:H28 strain RM12367-C1 cultured overnight on LBA supplemented with 800 ng/mL of mitomycin-C. The putative disulfide-bond-intact B-subunit of Stx2a is indicated. Bottom panel: MS/MS analysis of the putative B-subunit at *m/z* 7822 in the top panel. Fragment ions are indicated by their *m/z* and b- or y-type designations. The sequence of the Stx2a B-subunit is shown. A red asterisk indicates sites of polypeptide backbone cleavage that generate the corresponding fragment ions shown above (y-type) or below (b-type) the sequence. Acidic residues are highlighted in red and basic residues in blue. Cysteine residues are boxed, and a connector line symbolizes the intramolecular disulfide bond.

Figure 3 (top panel) shows the MS analysis of the *E. coli* O145:H28 strain RM12367-C1 cultured overnight on LBA supplemented with 800 ng/mL of mitomycin-C. The peak, at *m/z* 7822, is the [M+H]^+^ of the putative B-subunit of Stx2a. Its *m/z* is within the experimental error for MS linear mode. Unlike with STEC strain 493/89, we observe a number of bacterial host proteins at *m/z* 7277, 7712, 8331, 9070, 9747, 10,473, 15,751, and 18,175, as well as MALDI matrix adduct protein ions at *m/z* 9279 and 9956. The protein ion at *m/z* 7712 (i.e., YahO protein) can be particularly challenging, as it fragments efficiently and is near in *m/z* to the B-subunit of Stx2. There are several options to address this issue. One option is to suppress YahO expression during induction/culturing by optimizing the antibiotic concentration to maximize toxin expression and minimize host protein expression. Another option is to limit co-extraction of YahO during sample preparation by avoiding lysis of the bacterial cells so that only cells that are antibiotic-induced release proteins. Finally, it is possible to filter out (as much as possible) the YahO protein ion by narrowing the TIS during MS/MS. Although ions that are near in *m/z* will have ion packets that have a certain amount of overlap, it is possible to significantly reduce the contaminant ions by asymmetric narrowing of the TIS (although a certain amount of target ion loss is unavoidable).

Figure 3 (bottom panel) shows MS/MS of the protein ion at *m/z* 7822, using a TIS isolation of window of –50 Da/+75 Da. The narrower window on the low *m/z* side is to eliminate any contribution of YahO fragment ions from the MS/MS experiment. As the sample was not treated for disulfide-bond-reduction, fragmentation of the metastable B-subunit shows the characteristic fragment-ion triplets associated with the lasso-loop disulfide-bond-intact structure. For example, fragment-ion triplets (e.g., *m/z* 5078.7, 5112.3, and 5144.8) are the result of polypeptide backbone cleavage between the two cysteine residues and symmetric (S···S) and asymmetric cleavage (-SSH and HSS-) of the disulfide bond. The most prominent fragment ions were the result of polypeptide backbone cleavage on the C-terminal side of aspartic acid (D) and glutamic acid (E) residues, as discussed previously. The amino acid sequence is shown above. There are three distinct sets of fragment-ion triplets, resulting from symmetric and asymmetric cleavage of the disulfide bond, as well as polypeptide backbone cleavage on the C-terminal side of three aspartic acid residues (y_46_, y_53_ and y_54_), each located between the two cysteine residues. Polypeptide backbone cleavage not between the cysteine residues generates fragment ions without a triplet (b_57_ and b_64_). Although top-down proteomic analysis was performed on MS/MS of the disulfide-bond-reduced B-subunit, MS/MS of the disulfide-bond-intact B-subunit is useful, as it confirms the presence of the intramolecular disulfide bond, which is a critical post-translational modification (PTM) important to the secondary structure of the B-subunit. Obtaining such secondary structure information by bottom-up proteomic analysis is not possible, because the protein is reduced, alkylated, and digested, resulting in loss of this information.

**Figure 4:**
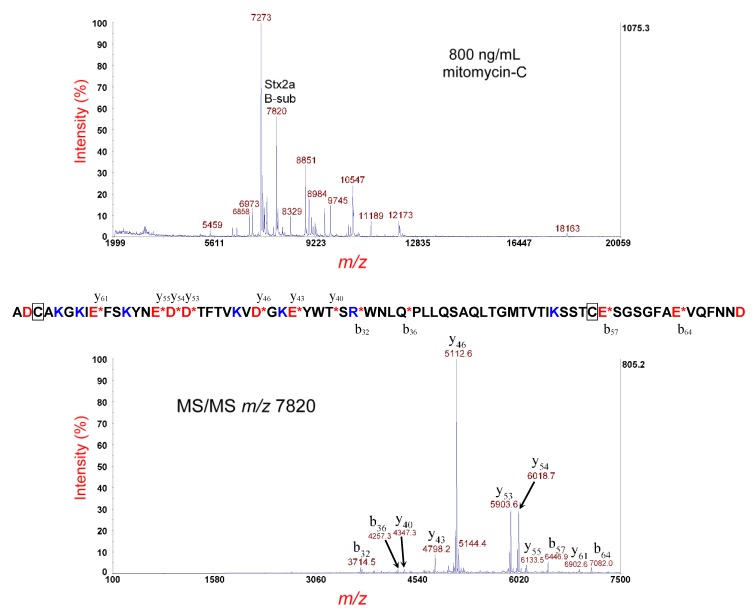
Top panel: MS analysis of *E. coli* O145:H28 strain RM12367-C1 cultured overnight on LBA supplemented with 800 ng/mL of mitomycin-C and disulfide-reduced. The putative disulfide-reduced B-subunit of Stx2a is indicated. Bottom panel: MS/MS analysis of the putative B-subunit at *m/z* 7820 in the top panel. Fragment ions are indicated by their *m/z* and b- or y-type designations. The sequence of the Stx2a B-subunit is shown. A red asterisk indicates sites of polypeptide backbone cleavage that generate the corresponding fragment ions shown above (y-type) or below (b-type) the sequence. Acidic residues are highlighted in red and basic residues in blue. Cysteine residues are boxed.

Figure 4 (top panel) shows MS analysis of the same strain cultured under the same conditions; however, the sample supernatant was disulfide-reduced prior to MS analysis. A peak at *m/z* 7820 is the [M+H]^+^ of the putative disulfide-reduced B-subunit of Stx2a. The theoretical average mass of the disulfide-bond-reduced B-subunit is 7817.64 Da; thus, the *m/z* is within experimental error for MS linear-mode analysis. The putative reduced B-subunit at *m/z* 7820 was isolated and fragmented by MS/MS-PSD, and the resulting spectrum is displayed in the Figure 4 (bottom panel). Once again, in contrast with the disulfide-bond-intact structure, a much simpler and interpretable MS/MS spectrum is obtained. Prominent fragment ions are labeled by *m/z* and b- or y-type designation. The most prominent fragment ions are the result of polypeptide backbone cleavage on the C-terminal side of D-residues (y_46_, y_53_, and y_54_), with the lesser abundant fragment ions resulting from cleavage on the C-terminal side of E-residues. All fragment ions possess the single arginine residue (R) of the B-subunit sequence, which suggests that the ionizing proton is sequestered at the R-residue, although there are six lysine residues (K) also present. The gas phase basicity (GB) of R-residue (237.0 kcal/mol) [30], the highest of any amino acid, may explain the preferential sequestration of the proton at this location. Finally, b_57_ and b_64_, which are very prominent in the disulfide-intact structure, are far less abundant in the disulfide-reduced structure, due, presumably, to differences in energy redistribution in their gas-phase structures. Top-down proteomic analysis using our in-house proteomic software identified this protein as the type/subtype: Stx2a with a *p*-value of 4.8 × 10–15 and a percentage of matched fragment ions: 41.67. The results were consistent with genomic sequencing.

### 3.3. Analysis of E. coli O145:H28 Strain RM14496-C1

**Figure 5:**
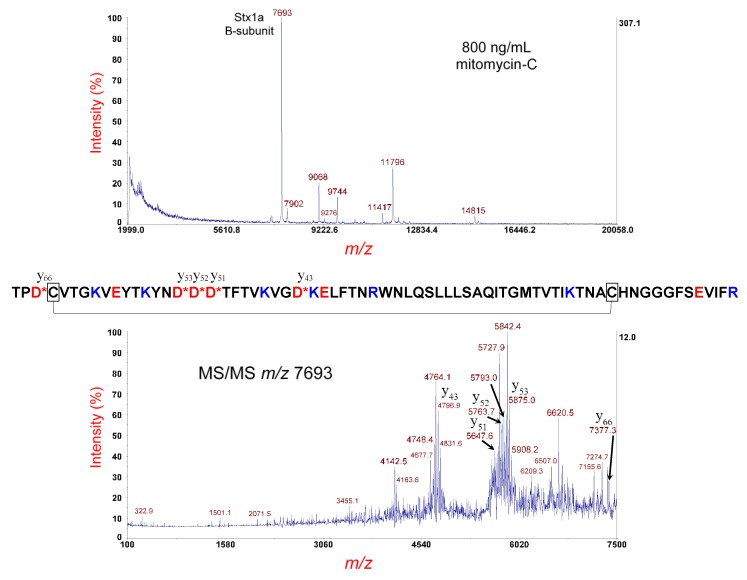
Top panel: MS analysis of *E. coli* O145:H28 strain RM14496-C1 cultured overnight on LBA supplemented with 800 ng/mL of mitomycin-C. The putative disulfide-bond-intact B-subunit of Stx1a is indicated. Bottom panel: MS/MS analysis of the putative B-subunit at *m/z* 7693 in the top panel. Fragment ions are indicated by their *m/z* and b- or y-type designations. The sequence of the Stx1a B-subunit is shown. A red asterisk indicates sites of polypeptide backbone cleavage that generate the corresponding fragment ions shown above (y-type) or below (b-type) the sequence. Acidic residues are highlighted in red and basic residues in blue. Cysteine residues are boxed, and a connector line symbolizes the intramolecular disulfide bond.

Figure 5 (top panel) shows the MS analysis of the *E. coli* O145:H28 strain RM14496-C1 cultured overnight on LBA supplemented with 800 ng/mL of mitomycin-C. The peak at *m/z* 7693 is the [M+H]^+^ of the putative B-subunit of Stx1a. The theoretical average mass of the disulfide-bond-intact (oxidized) B-subunit is 7688.66 Da. The difference in mass between the observed and theoretical value is within experimental error for MS linear-mode analysis (1000 ppm). Bacterial and prophage proteins are also detected at *m/z* 9068, 9744, 11,417, and 14,815. The peak at *m/z* 11,796 is residual calibrant (AlkTrx) used for MS/MS calibration. Matrix adduct peaks are detected at *m/z* 7902 and 9276.

The putative Stx1a B-subunit ions at *m/z* 7693 were isolated and fragmented by MS/MS-PSD, which is shown in Figure 5 (bottom panel). The MS/MS spectrum is quite complex due to polypeptide backbone cleavage on the C-terminal side of three adjacent aspartic acid (D) residues in the B-subunit sequence, as well as symmetric and asymmetric cleavage of the disulfide bond (i.e., fragment-ion triplets). Prominent fragment ions are identified by their *m/z* and b- and y-type designation. The amino acid sequence is shown above. All but one of the D-residues are located between the two cysteine residues of the intramolecular disulfide bond. Like the B-subunit of Stx2, polypeptide backbone cleavage that occurs between the two cysteine residues generates a fragment-ion triplet. For example, the y_43_ fragment ion at *m/z* 4796.9 is flanked by fragment ions at *m/z* 4764.1 and 4831.6 that are the result of asymmetric cleavage of the disulfide bond, with a spacing of ±33 *m/z* on either side of the *m/z* of y_43_. Similar flanking fragment ions are observed for: y_51_, y_52_, and y_53_. Fragment-ion triplets typically reduce the signal-to-noise (S/N) as the intensity of the precursor ion is distributed over a larger number of fragment ions for a given polypeptide backbone cleavage site. In consequence, top-down proteomic analysis is performed on the disulfide-reduced B-subunit, where the S/N is typically improved, as there are fewer fragmentation channels.

**Figure 6:**
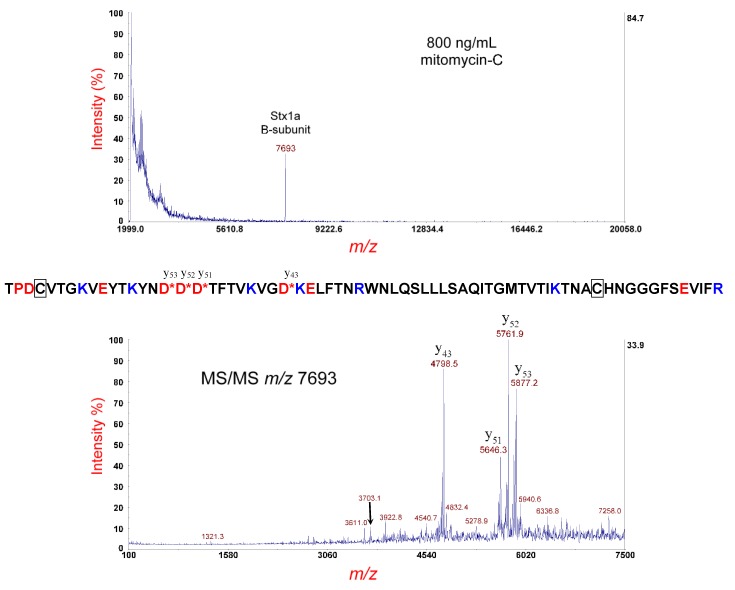
Top panel: MS analysis of *E. coli* O145:H28 strain RM14496-C1 cultured overnight on LBA supplemented with 800 ng/mL of mitomycin-C and disulfide-reduced. The putative disulfide-reduced B-subunit of Stx1a is indicated. Bottom panel: MS/MS analysis of the putative B-subunit at *m/z* 7693 in the top panel. Fragment ions are indicated by their *m/z* and y-type designations. The sequence of the Stx2a B-subunit is shown. A red asterisk indicates sites of polypeptide backbone cleavage that generate the corresponding fragment ions shown above (y-type) or below (b-type) the sequence. Acidic residues are highlighted in red and basic residues in blue. Cysteine residues are boxed.

Figure 6 (top panel) shows the MS analysis of the same strain cultured overnight on LBA supplemented with 800 ng/mL of mitomycin-C; however, the sample supernatant was disulfide-reduced prior to MS analysis. Once again, the peak at *m/z* 7693 is the [M+H]^+^ of the B-subunit of Stx1. Its theoretical average mass is 7690.67 Da and thus within experimental error for MS linear-mode. Although there is no apparent difference in *m/z* between the disulfide-bond-intact and the disulfide-bond-reduced B-subunits, the externally calibrated mass accuracy of MS linear-mode is not adequate to distinguish such slight differences in mass. The putative reduced B-subunit at *m/z* 7693 was isolated and fragmented by MS/MS-PSD, as shown in Figure 6 (bottom panel). Compared to the disulfide-bond-intact B-subunit, a far less congested MS/MS spectrum is obtained, as cleavage of the disulfide bond was eliminated as a fragmentation channel, and the polypeptide chain has a linear structure. The most prominent fragment ions are the result of polypeptide backbone cleavage on the C-terminal side of four (of the five) D-residues indicated in the sequence (shown above). The D-residue near the N-terminus does not generate a y_66_ fragment ion (unlike the disulfide-intact B-subunit), perhaps due to the aspartic acid effect rearrangement being less likely to occur at the termini of a linear chain.

Interestingly, all prominent fragment ions are y-type ions. No b-type fragment ions are detected. The reason for this is probably due to the ionizing proton being sequestered at either of the two arginine (R) residues in the sequence. Although protonation could occur at one of the five lysine residues (K) (or at the N-terminus), the gas phase basicity (GB) of arginine is 237.0 kcal/mol, whereas the GB of lysine is 221.8 kcal/mol [30], a difference of 15.2 kcal/mol that may give arginine a distinct advantage in sequestering the ionizing charge. In consequence, the complementary b-type fragment ions from cleavage on the C-terminal side of D-residues are not detected, because they have no R-residues. Along with polypeptide backbone cleavage, several small dissociative losses (NH_3_ and/or H_2_O) are also detected. In addition to charge sequestration, it is likely that the side-chain of arginine undergo facile dissociative loss of ammonia [31]. Top-down proteomic analysis of the MS/MS data, using our in-house proteomic software, identified this protein as the type/subtype: Stx1a with a *p*-value of 2.8 x 10–11 and the percentage of matched fragment ions: 35.00. The results were consistent with genomic sequencing.

## 4. Conclusions

We have analyzed three pathogenic STEC strains by antibiotic induction, MALDI-TOF-TOF mass spectrometry, and top-down proteomic analysis. Both Stx1 and Stx2 were successfully identified using top-down proteomic analysis of the B-subunit. DNA sequencing was consistent with top-down proteomic analysis. Although the disulfide-bond-reduced B-subunit was used for top-down identification, MS/MS of the disulfide-bond-intact B-subunit is useful, as it confirms the presence of this critical PTM. Two arginine residues present in the Stx1a B-subunit contribute to both charge sequestration, as well as dissociative losses of ammonia that increase the complexity of the MS/MS spectrum. The B-subunit of Stx2a has only one arginine residue in its sequence, and, although it too appears to sequester the ionizing proton, there appear to be fewer small dissociative losses compared to Stx1a, due presumably to there being only one arginine present in its sequence.

## Figures and Tables

**Table 1:** Strains used in this study.

Strain	Serotype	Stx Type/Subtype	Source	Reference	GenBank Accession #
493/89	O157:H-	*stx*2_a_	Human, Germany	[19,20]	
RM12367-C1	O145:H28	*stx*2_a_	Water, CA, USA	[21]	MK635342
RM14496-C1	O145:H28	*stx*1_a_	Feral pig, CA, USA	[21]	MK635343
